# Supplementary material for: Integration of multidimensional splicing data and GWAS summary statistics for risk gene discovery
Source: PLoS Genet. 2022 Jun 30;18(6):e1009814. doi: 10.1371/journal.pgen.1009814 (PMC9278751; doi:10.1371/journal.pgen.1009814)
Supplement: S1 Appendix — Note 1. Well-known trait-associated gene lists. Fig A. LocusZoom [82] plot for SNP rs9331888 near gene CLU. This SNP was identified as a significant sQTL (q-value = 2.7 × 10^−5^) in the ROSMAP study [17]. It is also nominally significant in the IGAP GWAS (p-value = 3.86 × 10^−5^). Fig B. LocusZoom [82] plot for SNP rs1991570 near gene PTK2B. This SNP was identified as a significant sQTL (q-value = 2.98 × 10^−13^) in the ROSMAP study [17]. It is also nominally significant in the IGAP GWAS (p-value = 3.67 × 10^−5^). Fig C. AD genes identified via splicing analysis using MSG that would have been missed from expression analysis using S-PrediXcan. Fig D. LDL-C genes identified via splicing analysis using MSG that would have been missed from expression analysis using S-PrediXcan. Fig E. Schizophrenia genes identified via splicing analysis using MSG that would have been missed from expression analysis using S-PrediXcan. Fig F. Power comparison between sCCA+ACAT with and without retraining the prediction model using elastic net in the first set of simulations. Fig G. Power comparison between SVD+χ2, GBJ, and ACAT tests using sCCA-generated splicing-CVs in the first set of simulations. Table A. Comparison of type I error for MSG using individual GWAS, MSG with GWAS summary statistics and reference genome of 400 and 5000 individuals in simulation. (PDF) [file pgen.1009814.s001.pdf]

Integration of multidimensional splicing data and GWAS summary statistics for risk gene discovery: supplementary notes

1. WELL-KNOWN TRAIT-ASSOCIATED GENE LISTS

A. AD genes

APOE, SORL1, GAB2, CR1, PICALM, CLU, CD33, ABCA7, ADAM10, CD2AP, BIN1, APOC1, TOMM40, INPP5D, PSEN2, EPHA1, APP, MTHFD1L, CNTNAP2, HLA-DRB1, CASS4, BCAM, ABCA1, PTK2B, MS4A6A, FRMD4A, BCL3, SLC24A4, GLIS3, FERMT2, PSEN1, TREM2, ZCWPW1, EXOC3L2, MS4A4A, ACE, APOC4, BZW2, SUCLG2, APOB, SCIMP, SCARB1, RELB, CRY2, PVRL2, CLASRP, ADAMTS4, MMP3, UBE2L3, PPP1R37, ECHDC3, TCF7L2, IL6R, MS4A2, LIPG, MAN2A1, MAPT, ALDH1A2, ABI3, LILRA5, CELF1, PLCG2, HMGCR, OARD1, APH1B, APOC2, OR4S1, STAT4, MS4A4E, PVR, MT-ND2, HS3ST1, CCR2, VASP, CYP8B1, BLOC1S3, PPP1R13L, NFIC, NKPD1, INSR, CNTNAP5, BCAS3, BCHE, BCL2, NME8, CLPTM1, CLNK, UBQLN1, CLMN, IL1B, TRAPPC6A, VSNL1, SORCS1, PPARG, IGSF23, CRH, PSMA1, CHRNB2, FBXL7, CHRNA7, SPON1, MYO16, CHRNA2, VLDLR, KIR3DL2, KIT, HLA-DRB5, BACE1, HLADRA, DSG2, CALHM1, RBFOX1, HFE, PILRA, LRP4, HARBI1, TFCP2, CBLC, DPP10, SYNJ1, CDC25B, ACP2, ACHE, PACSIN3, MADD, ZNF652, GSK3B, PFDN1, RIN3, MARK4, GRIN2A, PDGFRB, MAPK8IP1, GRIN3B, CCRL2, ECE1, SCN1A, HBEGF, CACNA1G, CEACAM16, MMP13, ESR1, ALDH5A1, PLAU, SCN8A, CACNA2D1, MMP12.

B. LDL-C genes

VAPB, APOE, SOAT1, LRPAP1, ADH1B, NPC1, PPARG, ANGPTL3, PCSK9, CYP27A1, KPNB1, CETP, LPIN3, NCEH1, TNKS, FADS3, LDLRAP1, OSBPL5, FADS1, VDAC1, PLTP, APOC2, LIPA, LPA, LIPC, SORT1, MYLIP, SCARB1, ABCB11, VDAC3, LRP2, APOB, APOH, TSPO, VAPA, LIPG, APOA4, APOC3, ALDH2, APOA1, NPC2, LRP1, LDLR, APOC1, STARD3, FADS2, CD36, ABCG5, ABCG8, STAR, APOA2, ABCA1, VDAC2, ANGPTL4, SOAT2, CYP7A1, IRF2BP2, LPL, LCAT.

C. Schizophrenia genes

IRAK4, CYC1, CHI3L1, FLJ46321, ATXN3, DENND1A, S100A12, ARAF, BICD2, DLG3, NRG3, DISC1, KCNH2, GRM3, ZNF804A, ERBB4, DRD2, AS3MT, SNX19, ARL6IP4, APOPT1, CYP2D6.

2. ADDITIONAL FIGURES AND TABLES

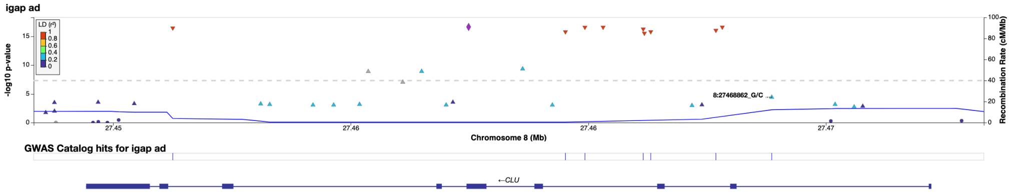

Fig. A. LocusZoom [1] plot for SNP rs9331888 near gene *CLU*. This SNP was identified as a significant sQTL (q-value $= 2.7 \times 10^{-5}$) in the ROSMAP study [2]. It is also nominally significant in the IGAP GWAS (p-value $= 3.86 \times 10^{-5}$).

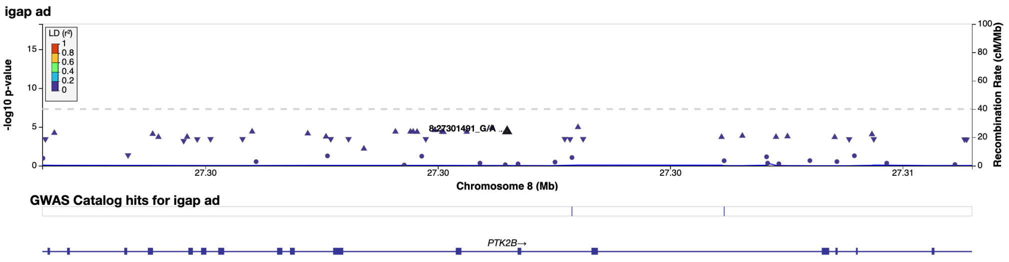

Fig. B. LocusZoom [1] plot for SNP rs1991570 near gene *PTK2B*. This SNP was identified as a significant sQTL (q-value $= 2.98 \times 10^{-13}$) in the ROSMAP study [2]. It is also nominally significant in the IGAP GWAS (p-value $= 3.67 \times 10^{-5}$).

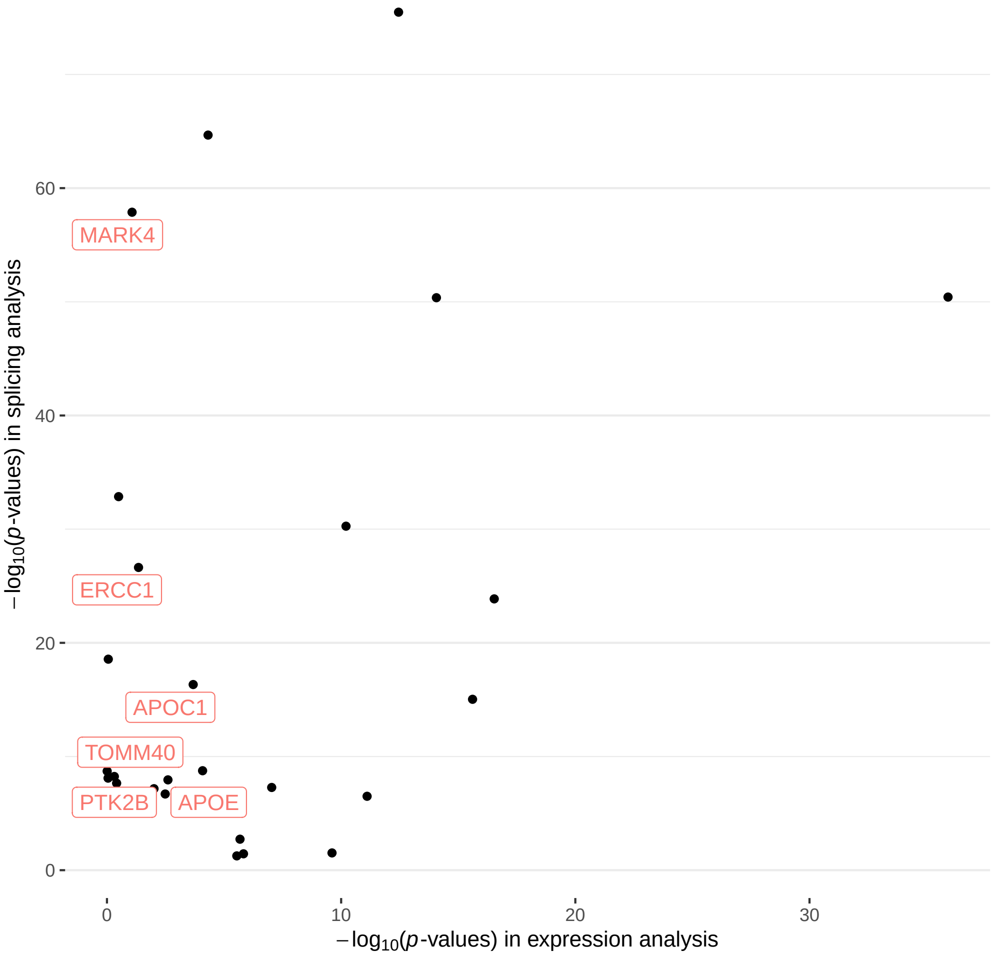

Fig. C. AD genes identified via splicing analysis using MSG that would have been missed from expression analysis using S-PrediXcan.

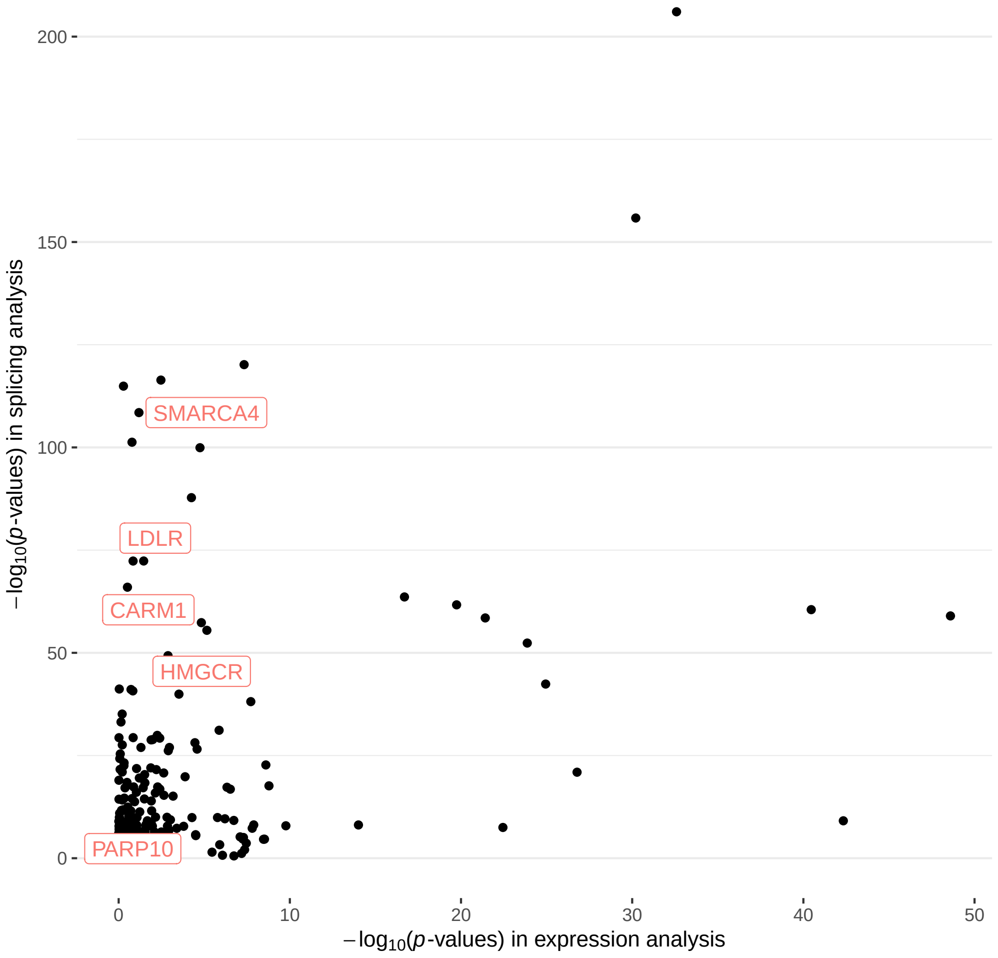

Fig. D. LDL-C genes identified via splicing analysis using MSG that would have been missed from expression analysis using S-PrediXcan.

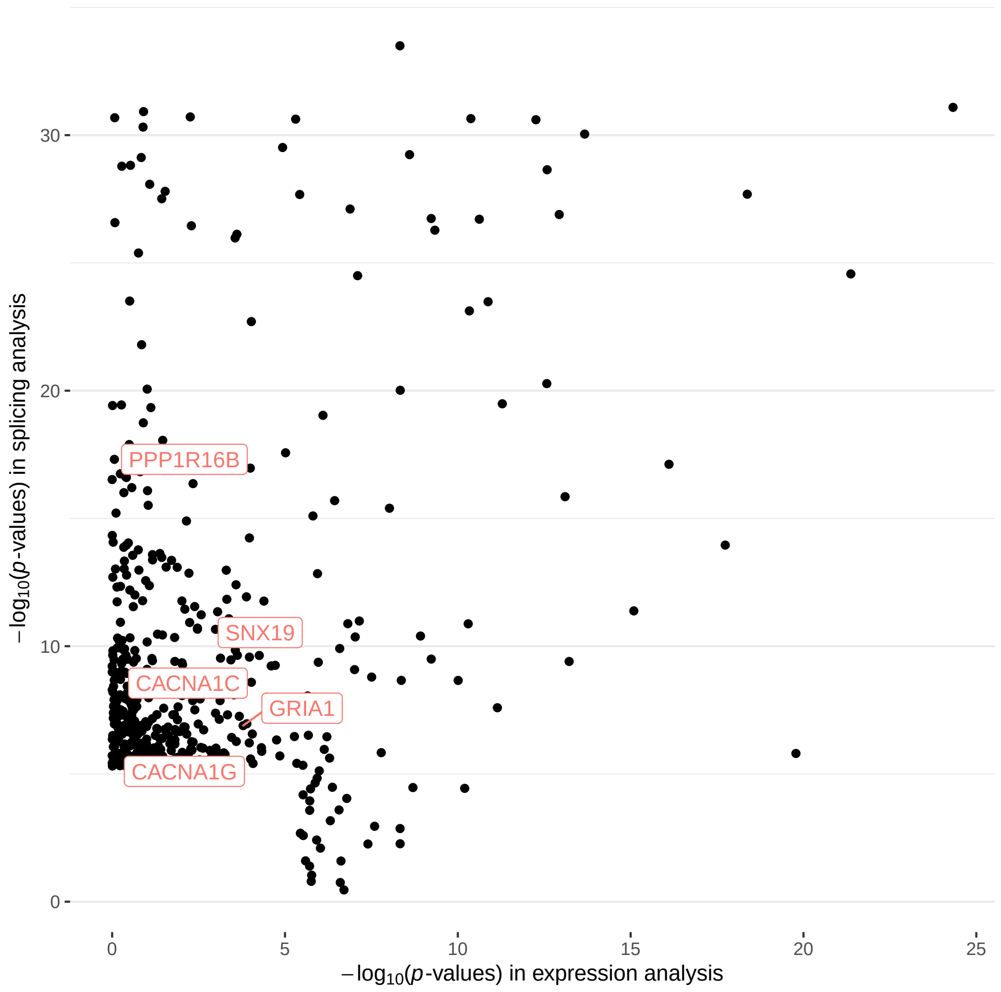

Fig. E. Schizophrenia genes identified via splicing analysis using MSG that would have been missed from expression analysis using S-PrediXcan.

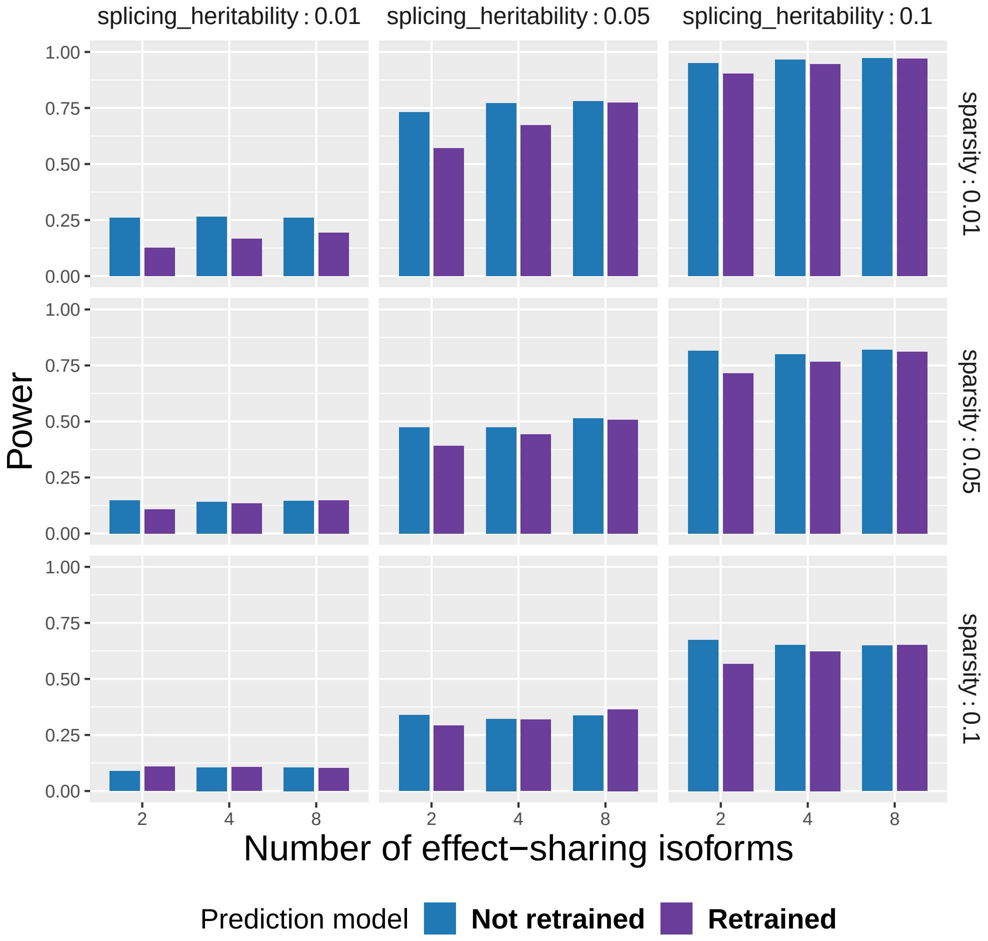

Fig. F. Power comparison between sCCA+ACAT with and without retraining the prediction model using elastic net in the first set of simulations.

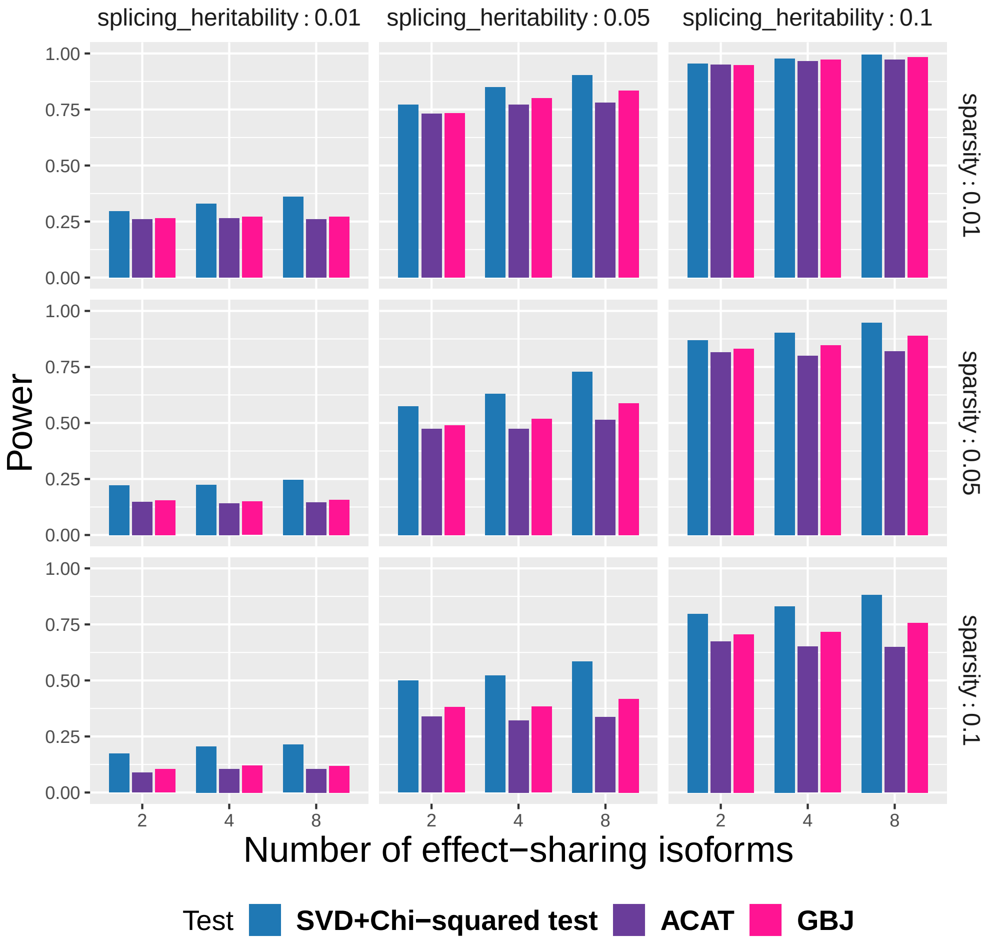

Fig. G. Power comparison between SVD+χ^2, GBJ, and ACAT tests using sCCA-generated splicing-CVs in the first set of simulations.

Table A. Type I error rates for MSG using individual-level GWAS data (MSG_{IND}), summary-level GWAS data with a LD reference panel of 400 individuals (MSG_{REF400}), and summary-level GWAS data with a LD reference panel of 5,000 individuals ($MSG_{REF5000}$).

Sharing	Sparsity	h_c^2	MSG_{IND}	MSG_{REF400}	$MSG_{REF5000}$
2	0.01	0.01	0.048	0.059	0.049
	0.01	0.05	0.050	0.059	0.050
	0.01	0.10	0.047	0.058	0.049
	0.05	0.01	0.051	0.061	0.051
	0.05	0.05	0.049	0.059	0.051
	0.05	0.10	0.050	0.060	0.052
	0.10	0.01	0.049	0.060	0.050
	0.10	0.05	0.052	0.063	0.052
	0.10	0.10	0.048	0.061	0.052
4	0.01	0.01	0.050	0.060	0.050
	0.01	0.05	0.052	0.061	0.051
	0.01	0.10	0.046	0.057	0.048
	0.05	0.01	0.050	0.059	0.051
	0.05	0.05	0.048	0.057	0.047
	0.05	0.10	0.047	0.060	0.051
	0.10	0.01	0.045	0.058	0.012
	0.10	0.05	0.050	0.060	0.047
	0.10	0.10	0.053	0.063	0.051
8	0.01	0.01	0.051	0.060	0.053
	0.01	0.05	0.051	0.059	0.050
	0.01	0.10	0.050	0.060	0.049
	0.05	0.01	0.046	0.055	0.047
	0.05	0.05	0.049	0.060	0.050
	0.05	0.10	0.050	0.060	0.050
	0.10	0.01	0.047	0.056	0.048
	0.10	0.05	0.049	0.059	0.049
	0.10	0.10	0.054	0.066	0.054

Note: Type I error was computed as the proportion of significant genes under the p-value cutoff of 0.05. Each entry is based on 20,000 replicates. The total number of splicing events is 10.

REFERENCES

1. R. J. Pruim, R. P. Welch, S. Sanna, T. M. Teslovich, P. S. Chines, T. P. Gliedt, M. Boehnke, G. R. Abecasis, and C. J. Willer, "Locuszoom: regional visualization of genome-wide association scan results," Bioinformatics **26**, 2336–2337 (2010).
2. T. Raj, Y. I. Li, G. Wong, J. Humphrey, M. Wang, S. Ramdhani, Y.-C. Wang, B. Ng, I. Gupta, V. Haroutunian *et al.*, "Integrative transcriptome analyses of the aging brain implicate altered splicing in alzheimer's disease susceptibility," Nat. genetics **50**, 1584–1592 (2018).
